# Test-retest reliability and symptom association of personalized depression TMS targets: A comparative study of refined seed-based (RSA) and hierarchical clustering (HCA) approaches

**DOI:** 10.1016/j.neurot.2026.e00884

**Published:** 2026-03-12

**Authors:** Hui Zhou, Yanmeng Bao, Jiasheng Xu, Dan Wang, Fengji Geng, Wanjun Guo, Yuzheng Hu

**Affiliations:** a The State Key Lab of Brain-Machine Intelligence, Zhejiang University, Hangzhou 310058, China; b Department of Psychology and Behavioral Sciences, Zhejiang University, Hangzhou 310058, China; c Department of Endocrinology, Children's Hospital of Zhejiang University, School of Medicine, National Clinical Research Center for Child Health, Hangzhou 310058, China; d Department of Curriculum and Learning Sciences, College of Education, Zhejiang University, Hangzhou 310007, China; e Affiliated Mental Health Center & Hangzhou Seventh People's Hospital, School of Brain Science and Brain Medicine, Zhejiang University School of Medicine, Hangzhou 310058, China; f MOE Frontiers Science Center for Brain Science & Brain-Machine Integration, Zhejiang University, Hangzhou 310058, China; g Nanhu Brain-Computer Interface Institute, Hangzhou 311121, China

**Keywords:** Personalized TMS targeting, Depression, Neuromodulation

## Abstract

Personalized transcranial magnetic stimulation (TMS) targeting holds promise for improving depression treatment, but its clinical translation is hindered by limited open-source implementation and systematic comparisons of target reproducibility and clinical relevance. We implemented two leading personalized TMS-target generating approaches, namely refined seed-based (RSA) and hierarchical clustering (HCA) algorithms, and compared them on (1) test-retest reliability of derived targets, and (2) association of target-sgACC connectivity with depressive symptoms. Using resting-state fMRI data from healthy and depressed individuals, spatial reliability was quantified via inter-run Euclidean distances, and clinical relevance was assessed through correlations between depression severity and functional connectivity of targets with sgACC. Effects of global signal regression (GSR) were also evaluated. The results showed that RSA produced targets in more superior and postrior part of DLPFC and demonstrated significantly higher test-retest reliability than HCA (smaller inter-run Euclidean distances). Further, RSA-derived target-sgACC connectivity correlated positively with depression severity, which was absent in HCA-derived targets. In addition, GSR improved spatial reliability for RSA but not HCA. Our results indicate that RSA exhibits superior test-retest reliability and symptom association compared to HCA, yet large-scale clinical trials are warranted to determine which approach yields superior therapeutic efficacy, and open-sourced implementation may accelerate clinical adoption.

## Introduction

Transcranial Magnetic Stimulation (TMS) is an established non-invasive brain stimulation technique for treating major depressive disorder (MDD) and other psychiatric conditions [[1], [2], [3]]. By inducing electrical currents, TMS modulates neural activity, offering therapeutic alternatives for patients resistant to traditional pharmacotherapy and psychotherapy [4,5]. Among various protocols, high-frequency excitatory repetitive TMS (rTMS) targeting the left dorsolateral prefrontal cortex (DLPFC) for MDD treatment has been approved by the U.S. Food and Drug Administration (FDA) and endorsed by clinical guidelines [6], yet treatment outcomes remain variable.

One key challenge in TMS treatment lies in identifying the optimal stimulation site within the DLPFC, a functionally heterogeneous region lacking clearly defined anatomical boundaries. Traditional TMS localization approaches (e.g. 5-cm rule, EEG F3 localization) suffer from poor spatial precision [7,8], inter-operator variability, and suboptimal clinical outcomes [9] – typically yielding response rate ≤40 % and remission rates ≤20 % [10]. To overcome these limitations, the anti-correlation between DLPFC stimulation sites and the subgenual anterior cingulate cortex (sgACC) has been identified as a key biomarker associated with antidepressant efficacy [11], suggesting that individualized targeting based on intrinsic functional connectivity (FC) could optimize treatment.

This neurobiological insight translates directly into improved clinical outcomes, as evidenced by converging findings from implementation studies. While direct comparisons between individualized and conventional targeting remain limited, closer proximity to individualized targets predicted better antidepressant response, with model projections suggesting a potential 14 % symptom improvement gain from precise targeting [12]. This aligns with empirical observations where algorithm-derived personalized TMS targets consistently displayed better response rate than conventional targeting [[13], [14], [15]]. The principle extends to invasive neuromodulation, as evidenced by an 81 % response rate for connectivity-guided DBS versus 41 % with anatomical targeting in depression [16]. Collectively, these studies highlight the value of target personalization while underscoring the need for methodological standardization.

**Fig. 1:**
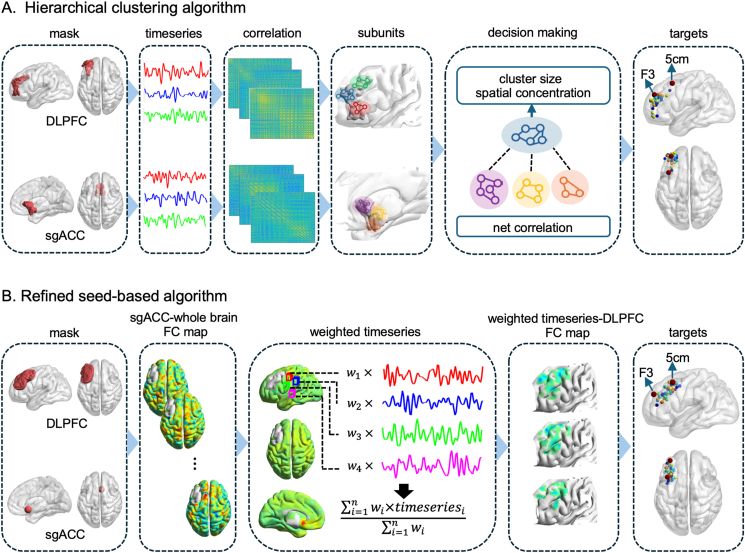
TMS Target generating flowchart of two algorithms. (A) The flowchart of the Hierarchical clustering algorithm. (B) The flowchart of the refined seed-based algorithm. The F3 location (at MNI coordinates [-35.5, 49.4, 32.4] [13]): and “5-cm” site (at MNI coordinates [-41, 16, 54] [21]): were represented in the figure for visualization.

To address this standardization gap and operationalize connectivity-based targeting, researchers have developed several algorithms for individualized TMS targeting, each demonstrating utility in small clinical cohorts [11,[17], [18], [19], [20]]. Among them, the refined seed-based algorithm (RSA) [21], and the hierarchical clustering algorithm (HCA) [13] have emerged as two leading methods. While the RSA optimizes target coordinates within a DLPFC mask using sgACC FC, the HCA identifies DLPFC coordinates based on cluster-level FC with sgACC. Preliminary studies have reported promising response/remission rates for both algorithms (RSA: 50–67 %; HCA: 78–90 %) [13,14,22], exceeding those typically achieved by traditional methods. However, these findings are based on small, non-comparative trials with significant differences in DLPFC and sgACC masks definition and strategies in determining target coordinates (Fig. 1).

These pervasive methodological differences present a fundamental obstacle to clinical translation and necessitates addressing two pivotal, yet unresolved, questions concerning the core properties of RSA and HCA: 1) reproducibility (how consistently do RSA and HCA derive the same target across repeated scans), and 2) symptom relevance (does target-sgACC FC robustly correlate with depression severity for both algorithms). This study aims to systematically address these questions by directly comparing the two TMS personalized targeting algorithms with two critical criteria: 1) test-retest reliability (Inter-run Euclidean distance between derived targets), and 2) depression symptom association (correlation between target connectivity profiles and depression severity). Additionally, we evaluated how global signal regression (GSR)—a controversal preprocessing step [23]—impacts both metrics. Through this systematic evaluation, we aim to provide an evidence-based framework for algorithm selection in personalized TMS, prioritizing reproducibility and symptom relevance to enhance the precision of individualized TMS protocols for depression treatment (The study was pre-registered at https://aspredicted.org/gkx3-6qk9.pdf. Pre-register number: AsPredicted #190958). The open-source implementation of the algorithms seeks to accelerate clinical adoption and collaborative refinement.

## Materials and methods

### Datasets

To evaluate the personalized targeting reliability across algorithms, MRI data of 100 unrelated participants were selected from Human Connectome Project (HCP) [24]. Participants (aged 22–36 years; 46 males, 54 females) completed two resting-state fMRI sessions on consecutive days. Each session included two runs (14 min 33 s/run; 1200 vol each) with alternating phase-encoding directions (right-to-left and left-to-right). During scanning, participants maintained eyes-open fixation on a central crosshair against a dark background. Data were acquired on a customized Siemens Skyra 3T scanner using a gradient-echo echo-planar imaging (EPI) sequence, and imaging parameters were as follows: repetition time (TR) = 720 ms, echo time (TE) = 33.1 ms, flip angle = 52°, field of view (FOV) = 208 × 180 mm^2^, matrix = 104 × 90, slice thickness = 2.0 mm, 72 slices, isotropic voxel size = 2.0 mm, multiband acceleration factor = 8.

To evaluate the symptom association of different algorithms, we analyzed data from 51 participants with mild depression in the OpenNeuro dataset (accession number: ds002748) [25]. Participants (aged 19–55 years; 13 males, 38 females) underwent eyes-closed resting-state fMRI during a single session comprising 100 dynamic scans. The fMRI data were acquired on a Philips 3 T Ingenia scanner at the International Tomography Center in Novosibirsk. Functional T2∗-weighted EPI scans were collected with the following parameters: voxel size = 2 × 2 × 5 mm^3^, TR = 2500 ms, TE = 35 ms, 25 slices. Russian versions of Beck Depression Inventory (BDI) and Zung Self Rating Depression Scale (SDS) were conducted to assess the severity of depression symptoms.

The data used in the current study were publicly available online, and informed consent was obtained from participants in the original studies [24,26]. This study was approved by the Ethic Committee of Zhejiang University.

### Preprocessing

HCP data were preprocessed with HCP minimal preprocessing steps, which included [1] gradient distortion correction [2], motion correction [3], single spline resampling of EPI frames into 2 mm isotropic MNI space, and [4] intensity normalization. The data were then spatially smoothed with a full width at half maximum Gaussian kernel of 5 mm, followed by nuisance regression that incorporated 6 head motion parameters and their temporal depravities, as well as the first 5 principal components from the white matter and CSF regions. A 0.01–0.1 Hz band-pass filter was further applied.

For the OpenNeuro dataset, preprocessing followed a similar pipeline but incorporated slice timing correction to account for the longer repetition time (TR). All processing was performed locally on a dedicated workstation.

GSR, routinely used in previous personalized targeting studies [11,21] but still in debate [23,27], was implemented to investigate its effect on target test-retest reliability and symptom association.

### DLPFC and sgACC mask definition

Masks for the DLPFC and sgACC were generated following established literature protocols. For HCA, masks were derived from the Brodmann atlas using left BA46 for DLPFC and bilateral BA25 for sgACC (Fig. 1A), consistent with Cole et al. [13]. For the RSA, masks were constructed per Cash et al. [21]: the DLPFC mask combined four spheric ROIs (20 mm radius centered at BA9 [MNI −36, 39, 43], BA46 [MNI −44, 40, 29], the “5 cm” TMS site [MNI −41, 16, 54], and the F3 Beam site [MNI −37, 26, 49]) intersected with an AAL-template gray matter mask, while the sgACC mask comprised a 10 mm sphere centered at the coordinates [MNI 6, 16, −10] as illustrated in Fig. 1B.

### The implementation of HCA

As illustrated in Fig. 1A, the optimal target was determined following the methodology outlined by Cole et al. [13]. First, we computed pairwise Spearman correlations between voxel time-series within the DLPFC mask and converted this to a distance matrix (1 - correlation). Using hierarchical agglomerative clustering with spatial constraints, we partitioned the DLPFC into subunits through an iterative bottom-up process: each voxel initialized as a cluster, with spatially adjacent clusters having the smallest distance merged iteratively until the minimum inter-cluster distance exceeded 0.5. The same clustering algorithm was applied to the sgACC. The median values at each time within each subunit composed a “median time course”. Given a voxel in the subunit, if its time course showed the highest correlation with the median time course, its coordinate was used to represent the anatomic location of the subunit. Next, the correlation coefficients between the representative voxel's time series of each DLPFC subunit and that of each sgACC subunit were computed. The individualized DLPFC target coordinate was then determined in a three-parameters space: 1) a sum of correlation with sgACC weighted by cluster size (i.e. multiplying correlation coefficient by the number of voxels in correspoding sgACC subunit), 2) a spatial concentration of each DLPFC subunit (i.e. the voxel count divided by the mean Euclidean distance between voxel pairs within the subunit), and 3) the voxel number of each DLPFC subunit. All parameters were z-scored across all DLPFC subunits. The DLPFC subunit demonstrating the highest composite z-score (summed across all parameters) was designated as the optimal target, and its coordinates were selected as the target coordinates.

### The implementation of RSA

As illustrated in Fig. 1B, the optimal target was determined following the methodology outlined by Cash et al. [21]. First, a functional connectivity map between the sgACC and whole-brain voxels in MNI space was computed for each subject. A group-level sgACC connectivity map was generated by averaging individual maps across participants, creating a population-based weighting mask. Next, preprocessed fMRI data were multiplied by this weighting mask, and a sgACC representative signal was generated by averaging the resulting time series from all voxels outside the DLPFC mask. Within the DLPFC mask, voxel-wise correlation coefficients between this representative signal and time series of each voxel were computed. Voxels were ascendingly ranked and the top 50 % voxels were retained. Finally, the centroid of the largest contiguous cluster within these retained voxels was designated as the optimal target using AFNI's *3dClust* command. To evaluate the robustness of the algorithm, the retained voxels were selected from the top 10 %–100 % with 10 % increments (Supplementary Figs. S1–S3, Tables S1–S2).

### Evaluation of spatial discrepancy between HCA and RSA

To evaluate the spatial distribution of targets, we averaged the four targets derived from four runs with the HCA and RSA, respectively. We then quantified inter-algorithm discrepancy using the 3D Euclidean distance between the HCA-derived and RSA-derived targets for each participant. Directional discrepancies along each axis were evaluated through direct comparisons of Cartesian coordinate offsets (x, y, z) between paired RSA-HCA targets across participants, using permutation testing (10,000 iterations) with significance thresholds set at *p* < 0.0166 (Bonferroni-corrected for three comparisons). This approach comprehensively characterized the anatomical orientation of target localization differences between RSA and HCA.

### Evaluation of individual discriminability between HCA and RSA

A ratio concerning inter-individual and inta-individual target distance was calculated to characterize individual discriminability of personalized TMS targeting algorithms. Intra-individual distance was computed as the mean Euclidean distance across all pairwise combinations of run-specific targets (four runs per participant). The inter-individual distance was calculated as the Euclidean distance between corresponding run-derived targets across all participant pairs. The ratio was defined as the inter-individual distance divided by intra-individual distances for each participant. While a higher ratio indicate a better discriminability between individuals relative to within-individual variability, permutation testing (10,000 iterations) compared these ratios between RSA and HCA, with statistical significance thresholded at *p* < 0.05.

### Evaluation of test-retest reliability between HCA and RSA

Test-retest reliability was defined as the consistency with which RSA and HCA derived spatially stable targets across repeated scans, quantified through intra-individual spatial variability. This primary metric was operationalized as the standard deviation (SD) of 3D Euclidean distances between the target location identified in each individual run and the centroid (mean location) of the four targets for that participant. To characterize directional biases, we secondarily decomposed this variability into axis-specific components by calculating SDs of coordinate displacements along the x, y, and z dimensions. Smaller values for both the composite 3D SD and axis-specific SDs indicate higher reliability. Permutation tests were performed for the four comparisons between two algorithms (3D distance, x/y/z axes), with significance set at *p* < 0.0125 (Bonferroni-corrected).

### Decomposition of potential factors influencing test-retest reliability between HCA and RSA

Building upon the spatial variability assessment, we systematically dissected two potential confounding factors—acquisition day and phase-encoding direction—that may contribute to observed target localization inconsistencies. For acquisition day effect, we tested whether Euclidean distances between targets derived from same-day fMRI runs would be significantly smaller than those from different-day acquisitions, while simultaneously evaluating how this acquisition day effect differed between RSA and HCA algorithms. Parallel analyses addressed encoding direction effects by examining if targets sharing identical phase-encoding directions exhibited reduced spatial dispersion compared to orthogonally encoded counterparts, with algorithmic differences in encoding direction effects quantified through direct contrast.

To operationalize these comparisons, we defined Intra-day distance (IntraDD) and Inter-day distance (InterDD) to evaluate the effect of acquisition day on the target test-retest reliability:

(1) Intra-day distance (IntraDD)

IntraDD was calculated using resting-state fMRI data from two sessions collected on the same day, and the formula was as follows:

$$IntraDD=\left(Distance\left(Day1_{LR},Day1_{RL}\right)+Distance\left(Day2_{LR},Day2_{RL}\right)\right)/2$$

$Day1_{LR}$ represents the TMS target coordinates calculated from the resting-state data collected with left-to-right (LR) phase encoding on the first day, and Distance (A, B) refers to the Euclidean distance between target A and target B. A smaller IntraDD indicates higher intra-day reliability of the algorithm.

(2) Inter-day distance (InterDD)

InterDD was calculated using resting-state fMRI data from two sessions collected on different days, and the formula was as follows:

$$InterDD=\left(Distance\left(Day1_{LR},Day2_{RL}\right)+Distance\left(Day1_{RL},Day2_{LR}\right)\right)/2$$

A smaller InterDD indicates higher inter-day reliability of the algorithm.

In addition, we defined Intra-phase distance (IntraPD) and Inter-phase distance (InterPD) to evaluate the effect of encoding direction on the target test-retest reliability:

(1) Intra-phase distance (IntraPD)

$$IntraPD=\left(Distance\left(Day1_{LR},Day2_{LR}\right)+Distance\left(Day1_{RL},Day2_{RL}\right)\right)/2$$

(2) Inter-phase distance (InterPD)

$$InterPD=\left(Distance\left(Day1_{LR},Day2_{RL}\right)+Distance\left(Day1_{RL},Day2_{LR}\right)\right)/2$$

The main effect of algorithm (HCA vs. RSA), the main effect of acquisition day (IntraDC vs. InterDC), and their interactions were assessed via permutation tests. Each permutation test was iterated 10,000 times. A Bonferroni-corrected significance threshold of *p* < 0.0166 (α = 0.05/3) was applied to account for multiple comparisons. Differences in encoding-direction effects between RSA and HCA were evaluated in the same way.

### Evaluation of symptom association between HCA and RSA

To evaluate the symptom association of individualized TMS targets, we examined the association between target-sgACC FC and depressive symptom severity in a clinical population. First, using the algorithm-derived target coordinates as centroids, we constructed spherical ROIs with an 8-mm radius. Next, FC between each target ROI and all voxels within the sgACC was computed. To examine the clinical relevance of these connectivity patterns, we performed voxel-wise multiple linear regression analyses within the sgACC, where depression severity scores served as the dependent variable and age and sex were included as covariates. To account for multiple comparisons, small volume correction (SVC) was applied using the 3dClustSim tool in AFNI [28], with thresholds set at a voxel-wise *p*-value <0.001 and a minimum cluster size of 4 voxels (based on the group's average spatial smoothness parameters estimated via the mixed-model autocorrelation function, ACF). Significant sgACC clusters correlating with symptom severity were interpreted as evidence supporting the target's therapeutic potential.

## Results

### RSA-derived targets are localized in more superior and posterior parts of DLPFC compared to HCA, and show higher individual discriminability

**Fig. 2:**
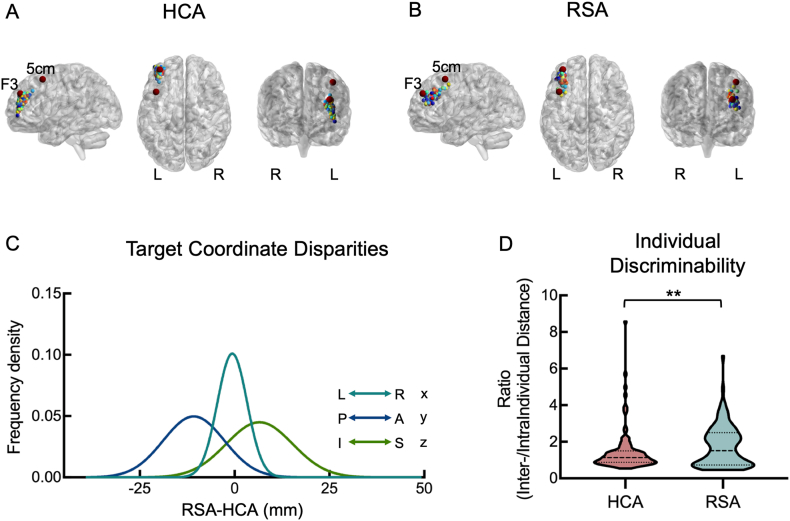
Characteristics of targets derived from the two algorithms, averaging across four runs of the HCP dataset. (A) The HCA-derived targets. (B) The RSA-derived targets at the 50 % ranking threshold. (C) The RSA targets located significantly more posterior and superior than HCA targets. (D) RSA targets displayed higher Inter-individual/intra-individual distance ratio than that of HCA. The horizontal line in the middle of the violin plots depicts the median and the lines above and below depict 25th and 75th quartiles. Abbreviations: HCA, hierarchical clustering algorithm; RSA, Refined seed-based algorithm. The red markers represent conventional targeting locations, including those determined by the standard “5-cm rule” [18,21] and the mean F3 coordinates derived from previous literature [13]. ∗∗*p* < 0.01.

The spatial distribution of personalized targets derived from two algorithms (RSA and HCA) is visualized in Fig. 2A and B. Specifically, target coordinates were averaged across four runs for each participant in the HCP dataset. For visualization clarity, the RSA-derived targets were presented by results obtained using the top 50 % ranking threshold and the full set of results (ranking from 10 % to 100 % voxels) is provided in Fig. S1. Between-algorithm comparison revealed a median spatial discrepancy (3d Euclidean distance) of 14.42 mm, with maximal divergence along the y-axis (Fig. 2C). The RSA targets were significantly more posterior (median $y_{RSA}-y_{HCA}$ = −11.30, *p* < 0.001) and superior (median $z_{RSA}-z_{HCA}$ = 6.23, *p* < 0.001) than HCA, with no significant difference along the x axis (median $x_{RSA}-x_{HCA}$ = −0.31, *p* = 0.352).

Statistical comparison on individual discriminability (i.e. the Inter-/Intra Individual ratio of target distances) demonstrated significant better discriminability in RSA algorithm than HCA (median $Ratio_{RSA}-Ratio_{HCA}$ = 0.38, *p* = 0.002, Fig. 2D), indicating greater inter-individual target separation relative to intra-individual variability. This finding underscores RSA's enhanced capacity to capture inter-individual neurobiological heterogeneity in DLPFC targeting.

### RSA demonstrates superior test-retest reliability, and both algorithms exhibit substantial individual variability in target reliability

**Fig. 3:**
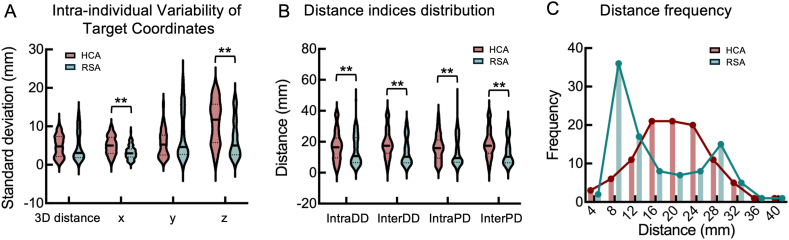
Target test-retest reliability of two algorithms. (A) Intra-individual variability of target coordinates, quantified by the standard deviation of 3D Euclidean distance and axis-specific (x, y, z) components. (B) Distance indices distribution of two algorithms. (C) Frequency histogram of averaged six-pair target distance. The dashed lines in the violin plot represent the upper quartile, median, and lower quartile, respectively. Abbreviations: IntraDD, intra-day distance; InterDD, inter-day distance; InterPD, inter-phase distance; IntraPD, intra-phase distance; HCA, Hierarchical clustering algorithm; RSA, Refined seed-based algorithm.∗∗*p* < 0.01.

Permutation tests (with *p* values < 0.0125 (0.05/4) considered as significant) revealed that HCA-derived targets exhibited significantly greater intra-individual variability than RSA for x-axis (median $SD_{x}^{HCA}-SD_{x}^{RSA}$ = 2.04, *p* < 0.001; Fig. 3A) and z-axis components (median $SD_{z}^{HCA}-SD_{z}^{RSA}$ = 6.72, *p* < 0.001; Fig. 3A), but not for 3D Euclidean distance (median $SD_{distance}^{HCA}-SD_{distance}^{RSA}$ = 1.65, *p* = 0.031, non-significant after correction) and y-axis (median $SD_{y}^{HCA}-SD_{y}^{RSA}$ = 0.65, *p* = 0.597).

Regarding how fMRI acquisition day and encoding-direction affect test-retest reliability of target, the RSA algorithm demonstrated a median value of 10.77 mm for IntraDD, 10.32 mm for InterDD, 9.41 mm for IntraPD, and 10.32 mm for InterPD (Fig. 3B, with a 50 % voxel threshold). Notably, these distance metrics exhibit a threshold-dependent decrease, with the median values declining to 8.90 mm (IntraDD), 8.39 mm (InterDD), 8.43 mm (IntraPD), and 8.39 mm (InterPD) at a 100 % threshold (full data across all thresholds are provided in Table S1). In comparison, the HCA algorithm shows substantially larger variability, with median values of 16.44 mm (IntraDD), 17.45 mm (InterDD), and 15.90 mm (IntraPD), and 17.45 mm (InterPD).

For the acquisition day effect on target test-retest reliability, permutation tests showed a significant main effect of algorithm (main effect of algorithm: HCA-RSA = 6.40 mm, *p* < 0.001; Fig. 3B), indicating a higher reliability for RSA regardless acquisition day. Neither main effect of acquisition day nor interaction was significant (main effect of acquisition day: *IntraDD*-*InterDD* = −0.28, *p* = 0.595; interaction: $\left(IntraDD_{HCA}-InterDD_{HCA}\right)-\left(IntraDD_{RSA}-InterDD_{RSA}\right)$ = −1.46, *p* = 0.547). Post-hoc tests suggest both IntraDD and InterDD were significantly smaller in RSA than HCA ($IntraDD_{RSA}-IntraDD_{HCA}$ = −5.67, *p* = 0.0089; $InterDD_{RSA}-InterDD_{HCA}$ = −7.13, *p* < 0.001). Similar results were found when considering algorithm and encoding-direction (*IntraPD* vs. *InterPD*) as two within-subject factors, that is, a significant main effect of algorithm was found, indicating a higher reliability of RSA regardless encoding direction (main effect of algorithm: HCA-RSA = 6.81, *p* < 0.001; Fig. 3B). Neither main effect of encoding-direction nor interaction effect was significant (main effect of encoding-direction: *IntraPD*-*InterPD* = −1.22, *p* = 0.025, non-significant after correction; interaction: $\left(IntraPD_{HCA}-InterPD_{HCA}\right)-\left(IntraPD_{RSA}-InterPD_{RSA}\right)$ = −0.65, *p* = 0.768). Post-hoc tests suggest both IntraPD and InterPD were significantly smaller in RSA than HCA ($InterPD_{RSA}-InterPD_{HCA}$ = −7.13, *p* < 0.001; $IntraPD_{RSA}-IntraPD_{HCA}$ = −6.48, *p* = 0.0012).

In short, only the main effects of the algorithm were identified in the permutation tests, suggesting that the RSA has higher reliability regardless of acquisiton day and encoding-direction during fMRI acquisition. As such, we averaged intra-individual distances for each algorithm to examine their distributions. The frequency diagram revealed that targets derived from the RSA and HCA both demonstrated great variability across individuals (Fig. 3C), with a higher proportion of individuals exhibiting smaller intra-individual distances (<10 mm) in RSA (49 %) compared to that in the HCA (14 %). To further characterize these “high variability” individuals, we performed supplementary analyses to determine whether inter-session target variability was driven by head motion or intrinsic image quality. Participants were stratified into “High Variability” and “Low Variability” groups based on the averaged six-pair target distance calculated for the targets identified by the RSA and HCA frameworks, respectively. The results revealed that “Low Variability” individuals (i.e., those with high reliability) in the RSA framework exhibited significantly higher mean temporal signal-to-noise ratio within the DLPFC (median difference = 2.52, *p* = 0.0204; Fig. S4). Regarding the HCA algorithm, no significant group differences (“Low Variability” relative to “High Variability”) were found across any evaluated metrics of head motion and image quality (Fig. S5). These findings suggest that RSA-based targeting is more sensitive to local signal stability, whereas HCA demonstrates greater robustness to both head motion and signal-to-noise ratio.

### GSR improves RSA reliability and individual discriminability but leaves HCA unchanged

Following GSR, the RSA algorithm exhibited pronounced reductions in distance metrics, whereas the HCA algorithm demonstrated relative stability. Specifically, for RSA, the median IntraDD decreased from 10.77 mm to 3.08 mm (*p* < 0.001), the median InterDD decreased from 10.32 mm to 3.11 mm (*p* < 0.001), the median IntraPD decreased from 9.41 mm to 3.16 mm (*p* < 0.001), and the InterPD decreased from 10.32 mm to 3.11 mm (*p* < 0.001) at the 50 % threshold (descriptive statistics for more thresholds are provided in Table S2). In contrast, HCA showed minimal changes in consistency measures: the median IntraDD changed from 16.44 mm to 16.02 mm (*p* = 0.835), the median InterDD changed from 17.45 mm to 16.35 mm (*p* = 0.239), the median IntraPD decreased from 15.90 mm to 14.80 mm (*p* = 0.330), and the median InterPD decreased from 17.45 mm to 16.35 mm (*p* = 0.239). As for the individual discriminability, GSR significantly improved the inter-/intra-individual ratio for the RSA (median $Ratio_{RSA\_GSR}-Ratio_{RSA\_noGSR}$ = 1.27, *p* < 0.001), but did not change for the HCA (median $Ratio_{HCA\_GSR}-Ratio_{HCA\_noGSR}$ = 0.05, *p* = 0.592). In addition, the proportion of individuals with smaller intra-individual distances (<10 mm) increased from 49 % to 95 % for RSA, but remained relatively stable (from 14 % to 17 %) for the HCA.

These findings highlight divergent sensitivities to GSR between the two algorithms, with RSA exhibiting marked metric improvements and HCA maintaining stability.

The higher test-retest reliability of RSA was also replicated in an independent clinical cohort with depression (Fig. S6). The results indicated that RSA exhibited superior test-retest reliability (lower SD and smaller averaged distance) compared to HCA when GSR was applied (Fig. S6). Without GSR, the results showed varying stability across axes, further highlighting the importance of preprocessing choices. In addition, when we reduce the data length of HCP from 1200 time points to fewer time points (e.g. 488), the results of target reliability remained unchanged regardless of whether GSR was performed (Fig. S6). These supplementary analyses demonstrate that the reliability advantage of RSA is not limited to high-quality, long-duration healthy cohorts but extends to clinical depression populations.

### RSA algorithm detects positive correlation between target-sgACC functional connectivity and depressive symptoms, absent in HCA

**Fig. 4:**
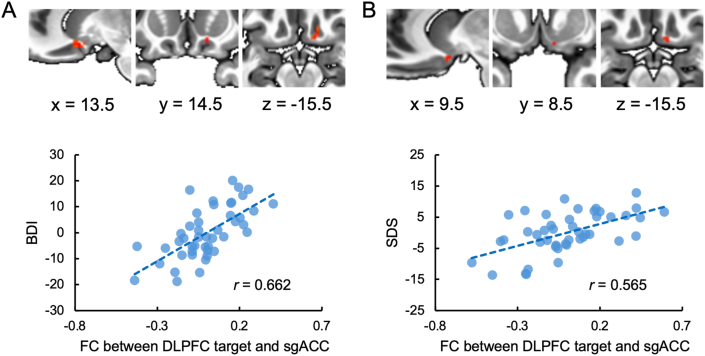
Correlations between depression symptoms and the functional connectivity between target and sgACC. (A) Regression results when using mean sgACC-whole brain FC map from OpenNeuro dataset as the weight map. (B) Regression results when using mean sgACC-whole brain FC map from HCP dataset as the weight map.

Regression results identified a cluster within the sgACC ROI, where functional connectivity between individualized DLPFC targets and this sgACC cluster was positively correlated with BDI scores at a threshold of 50 % in the RSA algorithm (Fig. 4A, please refer to Fig. S2 for more thresholds). This association was exclusive to RSA, as no comparable cluster was detected in HCA. When GSR was applied, the 50 % threshold correlation was diminished in RSA; notably, however, positive correlations emerged at 60 % and 70 % thresholds (Fig. S3).

### RSA generalizability confirmed using independent HCP dataset-derived sgACC connectivity as weight map

To further evaluate the applicability of the RSA, we utilized the mean sgACC-whole brain FC map from the HCP dataset as the weight map for the algorithm. Subsequently, we performed the same regression analysis using the new targets derived from the algorithm. The results revealed significant correlations between the FC of the DLPFC target-sgACC and depression symptoms (Fig. 4B). Critically, these associations remained intact when using the mean sgACC-whole brain FC from an independent, non-treatment cohort as the weight map, affirming the algorithm's robustness and broad applicability. After applying GSR, however, no significant cluster located at sgACC was found in the regression analysis for any threshold.

### HCA exhibits no significant correlation between target-sgACC functional connectivity and depression symptoms even with RSA-matched DLPFC search space

**Fig. 5:**
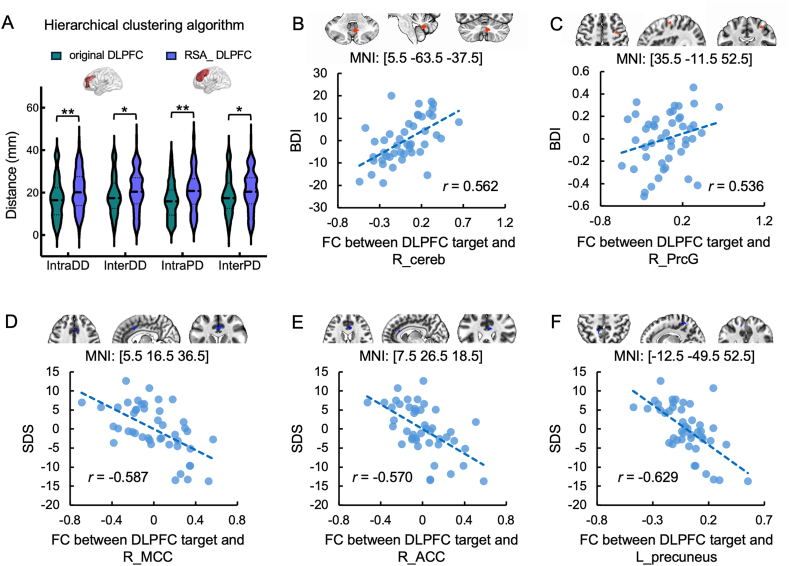
Target consistency and brain-behavior association when adopting large DLPFC mask for the hierarchical clustering algorithm. Abbreviations: R_cereb, right cerebellum; R_PrcG, right precentral gyrus; R_MCC, right middle cingulate cortex; R_ACC, right anterior cingulate cortex; L_ precuneus, left precuneus.∗ 0.01 ≤ *p* < 0.05, ∗∗*p* < 0.01.

In the preceding analysis, no significant relationship was observed between depression symptoms and target-sgACC FC for the HCA. Considering the disparity in search space between the two algorithms (a smaller DLPFC mask used for the HCA, focusing on the BA 46 region, versus a larger DLPFC mask for the RSA), we further examined whether the absence of a brain-behavior association could be attributed to this spatial constraint. Using the same DLPFC mask as the RSA, we observed a significantly increase in distance indexes ($IntraDD_{RSA\_DLPFC}-IntraDD_{original\_DLPFC}$ = 3.70, *p* = 0.0048; $InterDD_{RSA\_DLPFC}-InterDD_{original\_DLPFC}$ = 3.01, *p* = 0.016; $IntraPD_{RSA\_DLPFC}-IntraPD_{original\_DLPFC}$ = 4.93, *p* < 0.001; $InterPD_{RSA\_DLPFC}-InterPD_{original\_DLPFC}$ = 3.01, *p* = 0.016; *p* values < 0.0166 were considered significant, Fig. 5A); however, no significant correlation was identified between the FC of the DLPFC target-sgACC and depression symptoms. Whole-brain analysis revealed significant associations between DLPFC target FC and depression symptoms in several regions (*p* < 0.001, cluster size >18 according to whole-brain multiple comparison correction), including the right cerebellum, right precentral-gyrus, right middle cingulate cortex, right anterior cingulate cortex, and left precuneus (Fig. 5B–F). After applying GSR, no significant cluster located at sgACC was found in the regression analysis.

## Discussion

This study systematically compares two personalized TMS targeting approaches (RSA vs. HCA) in terms of target test-retest reliability and symptom association. Spatially, RSA localized DLPFC targets in significantly more posterior and superior subregions than HCA while demonstrating greater discriminability across individuals. Crucially,RSA exhibited superior test-retest reliability, evidenced by reduced intra-individual variability in target positioning across both intra-day and inter-day fMRI acquisitions and minimal sensitivity to acquisition encoding directions. Critically, only RSA-derived targets demonstrated a positive association between functional connectivity with the sgACC and depressive symptom severity; no such association was observed for HCA-derived targets. This brain-behavior relationship remained robust when validated using an independent sgACC connectivity template, highlighting the generalizability of RSA. GSR further enhanced RSA's test-retest reliability—an effect absent in HCA, though its impact on symptom association was threshold-dependent. Despite these advances, substantial interindividual variability in reliability persists across both methods, highlighting the practical challenges in achieving consistently precise individualized targeting.

The direct comparison between RSA and HCA provides a valuable opportunity to examine how distinct computational strategies address the challenge of personalized neuromodulation in depression. Although rooted in different mathematical frameworks, both algorithms share a common neurobiological foundation: the dysregulation of the sgACC–DLPFC circuit. RSA operates by identifying an optimal stimulation coordinate within a continuous connectivity gradient, emphasizing relational patterns between the seed and target regions. In contrast, HCA focuses on voxel-wise heterogeneity to parcellate the DLPFC into discrete functional subunits. These two approaches represent divergent methodological pathways toward the same therapeutic objective: identifying the DLPFC site that most effectively modulates sgACC hyperactivity. From a clinical perspective, comparing them helps clarify whether individualized targeting is better achieved through continuous mapping or discrete parcellation. Our study thus offers a meaningful reference for the selection of methodological approaches in clinical targeting.

High target test-retest reliability is essential for intervention fidelity, as inconsistent target localization across sessions would fundamentally undermine the premise of personalized neuromodulation. In repetitive TMS protocols—where treatment efficacy hinges on precisely engaging symptom-relevant circuits across multiple sessions—low reliability introduces uncontrolled variability, potentially diluting clinical outcomes. Our results indicated that RSA demonstrated superior spatial consistency, evidenced by significantly lower intra-individual variability and higher target test-retest reliability (i.e. smaller Intra/InterDD and Intra/InterPD) compared to HCA. This finding aligns with prior evidence from Cash et al. [21], which proposed that RSA has great target precision. At the individual level, the proportion of participants showing extremely low (<10 mm) inter-run target distances was substantially larger in the RSA group than in the HCA group. Nevertheless, substantial inter-individual variability in both algorithms suggests that single-session targeting may be insufficient for reliable individualized application for some participants.

While RSA demonstrates superior spatial stability (millimeter-level precision), it is also important to consider that TMS efficacy may also be governed by the functional consistency of the stimulated node. HCA, by identifying the functional centroid of a clustered region, may offer a form of “functional buffering.” In this context, HCA's lower spatial precision could, paradoxically, be a marker of its robustness against minor anatomical or physiological noise, ensuring the target remains within a functionally coherent network. Another significant practical advantage of HCA lies in its computational autonomy. Unlike RSA, HCA is not dependent on normative functional templates or group-level priors to refine target identification, making it particularly valuable in clinical settings where normative databases are unavailable or for patients whose brain architecture significantly deviates from standard templates. For these centers, HCA represents a crucial “entry-level” personalized targeting strategy that balances individualization with clinical feasibility.

The capacity to alleviate clinical symptoms by modulating symptom-relevant neural circuits through target stimulation constitutes another essential property of clinically effective personalized targeting algorithms. The DLPFC exhibits notable functional heterogeneity along a posterior-superior to anterior-inferior gradient. Specifically, posterior-superior subregions (e.g., BA9) demonstrate strong structural and functional connectivity with the anterior cingulate cortex (ACC), while anterior-inferior subregions (e.g., BA46) display weak ACC coupling [29]. This neuroanatomical organization explains our key comparative findings: RSA localized targets to posterior-superior DLPFC regions, where functional connectivity with the sgACC significantly correlated with depression severity. The observed positive correlation between DLPFC-sgACC functional connectivity and depressive symptom severity is, in fact, highly consistent with the established anti-correlation framework in which the DLPFC is hypothesized to have an inhibitory modulation over the sgACC to maintain a non-depressive state. Our finding that higher functional connectivity values track with increased symptom severity reconcile with the idea that a weakening or loss of this normative anti-correlation is a hallmark of the depressive state. Particularly, considering that our sample included both mild (i.e. BDI <19) and severe (BDI >28) individuals [30], the observation that lower connectivity values—representing more robust anti-correlated relationships—are associated with lower symptom scores further strengths the rationale for therapeutic intervention: targeting this specific node allows TMS to modulate the underlying dysregulation, with the goal of “restoring” the normative anti-correlation required for clinical remission. This supports the “DLPFC-sgACC” circuit hypothesis and highlights RSA's ability to identify symptom-relevant neuromodulation pathways. Reproducing this association using independent cohort-derived weight maps further strengthens RSA's clinical relevance while confirming its generalizability. In contrast, HCA-derived targets showed no significant association between functional connectivity with the sgACC and depressive symptoms. However, whole-brain analysis revealed that their connectivity with alternative regions—including the cerebellum, precentral gyrus, and middle cingulate cortex—did exhibit significant correlations with depression severity. This pattern suggests HCA engages distributed network mechanisms rather than discrete symptom-specific pathways. Consequently, RSA's seed-based approach enables focal modulation of established depression circuits (e.g., DLPFC-sgACC), while HCA's data-driven methodology may capture broader, multi-nodal networks with distinct clinical relevance.

The role of global signal regression (GSR) in resting-state fMRI remains debated [27]. While some studies advocate GSR for mitigating physiological artifacts [31,32], others caution against spurious negative correlations and neural signal distortion [33,34]. Our study showed that GSR further amplified RSA's advantages in target reliability while fundamentally preserving the depression-related sgACC connectivity association—despite altering its threshold dependency. However, GSR exerted no significant impact on either aspect in HCA. The differential impact of GSR on the performance of the RSA and HCA algorithms may underscore their distinct methodological foundations. RSA is fundamentally an extrinsic-reference approach that relies on an external sgACC-representative signal derived from a whole-brain weighting mask. In the absence of GSR, this reference signal is heavily contaminated by the global signal—a dominant source of non-neuronal variance. The global signal varies between sessions, and leads to reduced inter-session reliability. By removing this common-mode noise, GSR may effectively unmask the specific neural oscillations within the sgACC-DLPFC circuit, thereby significantly enhancing the spatial reproducibility of the RSA centroid. Conversely, HCA functions as an intrinsic-structure approach that defines subunits based on the similarity in connectivity with sgACC between voxels within the local DLPFC mask. Because the global signal affects voxels within a local region relatively uniformly, the relative similarity and topological branching within the hierarchical clustering tree remain largely invariant to global fluctuations. Furthermore, HCA's integration of geometric parameters, such as spatial concentration and cluster size, provides an additional layer of robustness against global baseline shifts, rendering it a topologically stable but less responsive alternative compared to the RSA.

In conclusion, our study provides a comprehensive comparison of two leading personalized TMS targeting algorithms. RSA demonstrated superior test-retest reliability compared to HCA, with its derived targets exhibiting a functional association with depressive symptoms. This positions RSA as a more reliable strategy for personalized targeting. However, “symptom association” do not directly equal to “clinical efficacy”, and its superior clinical efficacy remains to be verified in future randomized controlled trials. Moreover, the clinical implementation of these algorithms requires careful consideration of three key parameters: the masks adopted, preprocessing steps (especially GSR), and threshold selection. Of note, to mitigate the confounding effects of cross-site variability in scanning protocols and parameters, our study employed intra-dataset comparisons: reliability was assessed using the HCP dataset, while symptom association was evaluated within the OpenNeuro dataset. While this approach helps isolate algorithm-specific performance, how different scanners and sequence parameters affect the reliability and effectiveness of these two algorithms warrants future validation in large-scale, multi-platform longitudinal depression cohorts and randomized controlled trials.

## Author contributions

Hui Zhou is responsible for data analysis, draft writing and revision.

Yanmeng Bao is responsible for data collection and data analysis.

Jiasheng Xu is responsible for data analysis and result verification.

Dan Wang is responsible for data preprocessing.

Fengji Geng is responsible for project design and manuscript revision.

Wanjun Guo is responsible for conceptualization, resource support and manuscript revision.

Yuzheng Hu is responsible for research direction guidance, data analysis review and final version polishing.

## Data statement

The codes for RSA and HCA are available at: https://github.com/HuiiiZ/PersonalizedTargeting_NI.

## Declaration of competing interest

The authors declare that they have no known competing financial interests or personal relationships that could have appeared to influence the work reported in this paper.
