# Supplementary material for: Test-retest reliability and symptom association of personalized depression TMS targets: A comparative study of refined seed-based (RSA) and hierarchical clustering (HCA) approaches
Source: Neurotherapeutics. 2026 Mar 12;23(2):e00884. doi: 10.1016/j.neurot.2026.e00884 (PMC12996647; doi:10.1016/j.neurot.2026.e00884)
Supplement: Multimedia component 1 [file mmc1.docx]

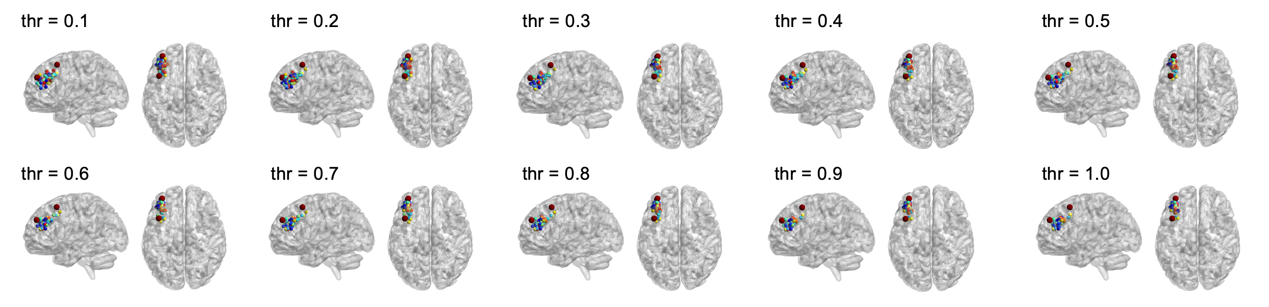


Figure S1. Target distribution for refined seed-based algorithm. The red markers represent conventional targeting locations, including those determined by the standard “5-cm rule” (Fox, Liu et al. 2013, Cash, Cocchi et al. 2021) and the mean F3 coordinates derived from previous literature (Cole, Stimpson et al. 2020).

Table S1. Descriptive statistics of target reliability index

|  | **IntraDD** | | | **InterDD/InterPD** | | | **IntraPD** | | |
| --- | --- | --- | --- | --- | --- | --- | --- | --- | --- |
|  | **median** | **min** | **max** | **median** | **min** | **max** | **median** | **min** | **max** |
| **HCA** |  |  |  |  |  |  |  |  |  |
|  | 16.44 | 1.41 | 38.67 | 17.45 | 1.41 | 40.19 | 15.90 | 2.73 | 37.62 |
| **RSA** |  |  |  |  |  |  |  |  |  |
| threshold=0.1 | 16.34 | 3.54 | 47.07 | 14.08 | 3.25 | 46.93 | 16.47 | 1.79 | 47.31 |
| threshold=0.2 | 14.01 | 2.62 | 50.57 | 13.29 | 2.76 | 55.75 | 14.48 | 2.96 | 55.67 |
| threshold=0.3 | 11.74 | 3.58 | 50.29 | 11.97 | 3.27 | 55.56 | 11.55 | 2.38 | 55.51 |
| threshold=0.4 | 11.45 | 2.95 | 49.08 | 11.13 | 2.74 | 48.91 | 10.59 | 1.55 | 47.23 |
| threshold=0.5 | 10.77 | 3.05 | 46.92 | 10.32 | 2.64 | 33.16 | 9.41 | 1.40 | 47.21 |
| threshold=0.6 | 9.84 | 2.45 | 46.98 | 9.78 | 2.20 | 36.20 | 8.76 | 1.30 | 47.26 |
| threshold=0.7 | 8.95 | 2.39 | 46.95 | 8.90 | 2.05 | 32.87 | 8.59 | 1.29 | 47.23 |
| threshold=0.8 | 8.95 | 2.37 | 43.07 | 9.10 | 2.02 | 37.87 | 8.47 | 1.18 | 43.18 |
| threshold=0.9 | 8.85 | 2.29 | 34.39 | 8.27 | 2.14 | 38.01 | 8.38 | 1.20 | 37.52 |
| threshold=1.0 | 8.90 | 2.26 | 34.39 | 8.39 | 2.12 | 45.62 | 8.43 | 1.23 | 46.14 |

Table S2. Descriptive statistics of target reliability index after GSR

|  | **IntraDD** | | | **InterDD/InterPD** | | | **IntraPD** | | |
| --- | --- | --- | --- | --- | --- | --- | --- | --- | --- |
|  | **median** | **min** | **max** | **median** | **min** | **max** | **median** | **min** | **max** |
| **HCA** |  |  |  |  |  |  |  |  |  |
|  | 16.02 | 2.41 | 41.45 | 16.35 | 3.15 | 41.43 | 14.80 | 3.86 | 40.22 |
| **RSA** |  |  |  |  |  |  |  |  |  |
| threshold=0.1 | 4.33 | 0.80 | 24.91 | 4.29 | 0.52 | 28.74 | 3.94 | 0.55 | 28.75 |
| threshold=0.2 | 3.63 | 0.99 | 20.65 | 3.96 | 0.92 | 28.09 | 3.42 | 0.48 | 28.08 |
| threshold=0.3 | 3.87 | 0.82 | 20.83 | 3.74 | 0.57 | 21.12 | 3.33 | 0.82 | 17.49 |
| threshold=0.4 | 3.36 | 0.79 | 20.38 | 3.38 | 0.53 | 20.54 | 3.30 | 0.63 | 17.37 |
| threshold=0.5 | 3.08 | 0.68 | 13.32 | 3.11 | 0.51 | 13.84 | 3.16 | 0.63 | 13.04 |
| threshold=0.6 | 2.88 | 0.71 | 12.50 | 2.93 | 0.42 | 13.85 | 2.91 | 0.59 | 12.96 |
| threshold=0.7 | 2.83 | 0.63 | 11.36 | 2.75 | 0.39 | 12.61 | 2.73 | 0.60 | 11.59 |
| threshold=0.8 | 2.84 | 0.81 | 10.36 | 2.82 | 0.83 | 11.84 | 2.70 | 0.59 | 11.94 |
| threshold=0.9 | 2.50 | 0.75 | 10.14 | 2.63 | 0.47 | 11.59 | 2.72 | 0.59 | 11.66 |
| threshold=1.0 | 2.50 | 0.68 | 10.49 | 2.60 | 0.48 | 11.48 | 2.60 | 0.56 | 11.55 |


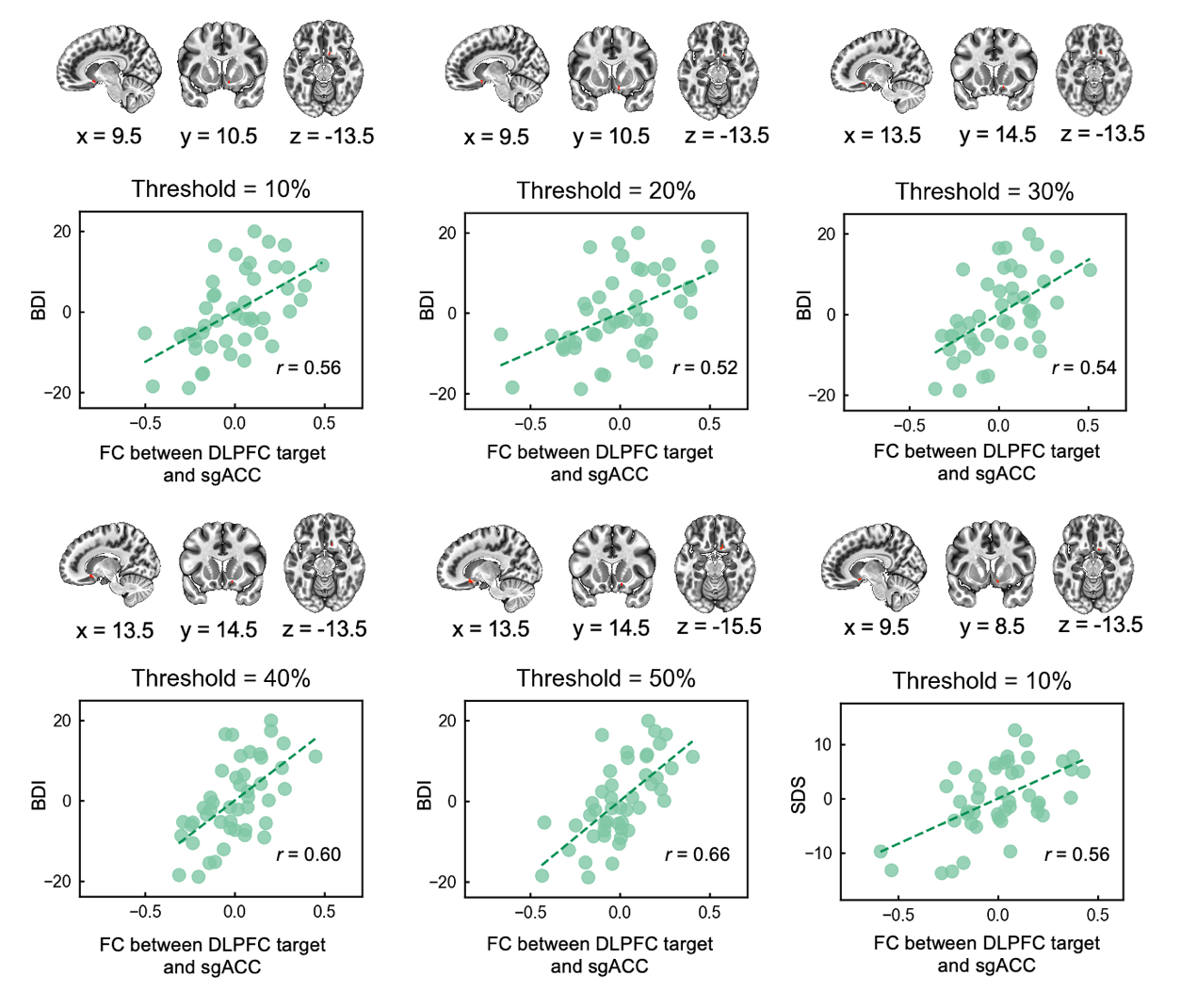


Figure S2. Correlations between depression symptoms and the functional connectivity between target and sgACC for refined seed-based algorithm.


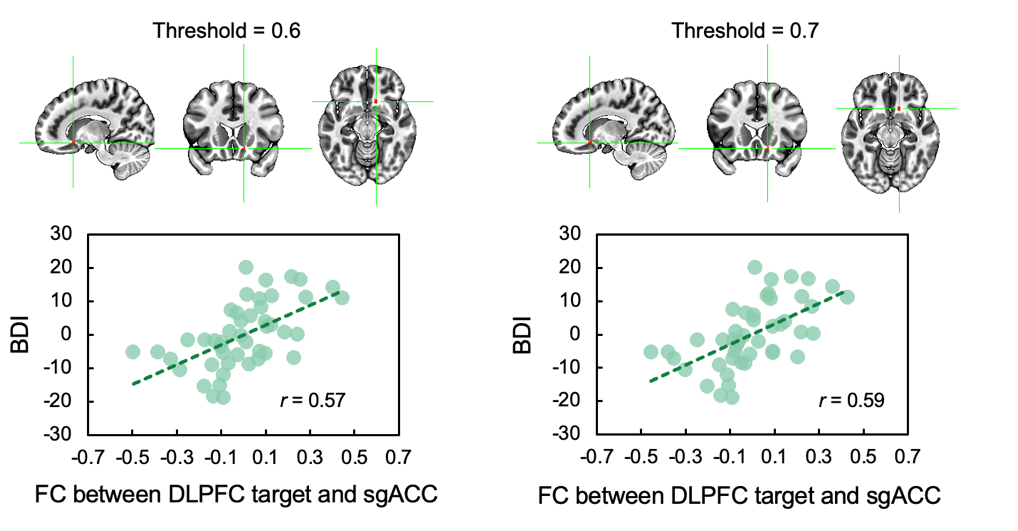


Figure S3. Correlations between depression symptoms and the functional connectivity between target and sgACC after global signal regression for refined seed-based algorithm.

**Supplementary Analysis 1: Investigation of Factors Influencing Inter-session Target Variability**

To investigate whether inter-session target variability was driven by physical head motion or intrinsic image signal quality, we conducted a series of supplementary analyses. Participants were stratified into “High Variability” and “Low Variability” groups based on the averaged six-pair target distance calculated for the targets identified by the RSA and HCA frameworks, respectively.

*Methods*

For each group, we evaluated two primary categories of confounding factors:

**Head Motion**: Quantified by the mean and variance of Frame-wise Displacement (FD) across all four fMRI sessions. This approach allowed for the assessment of both the overall magnitude of motion and its temporal stability.

**Image Signal Quality**: Quantified by the mean and variance of the temporal signal-to-noise ratio (tSNR) extracted from the left DLPFC (the target region) and the sgACC (the seed region) across all sessions.

Group comparisons were performed using non-parametric permutation tests (10,000 iterations). This method was chosen to provide robust statistical estimations given the non-normal distribution of target variability metrics.

*Results*

The analysis revealed distinct sensitivity profiles for the two algorithms. Within the RSA framework, participants in the “Low Variability” group (exhibiting higher targeting reliability) demonstrated significantly higher mean tSNR within the DLPFC compared to the “High Variability” group (median difference = 2.52, *p* = 0.0204; Figure S4). No significant group differences were observed for head motion metrics (mean FD or FD variance) or for the signal quality within the sgACC. In contrast, for the HCA algorithm, no significant group differences were found across any of the evaluated head motion or signal quality metrics (Figure S5).


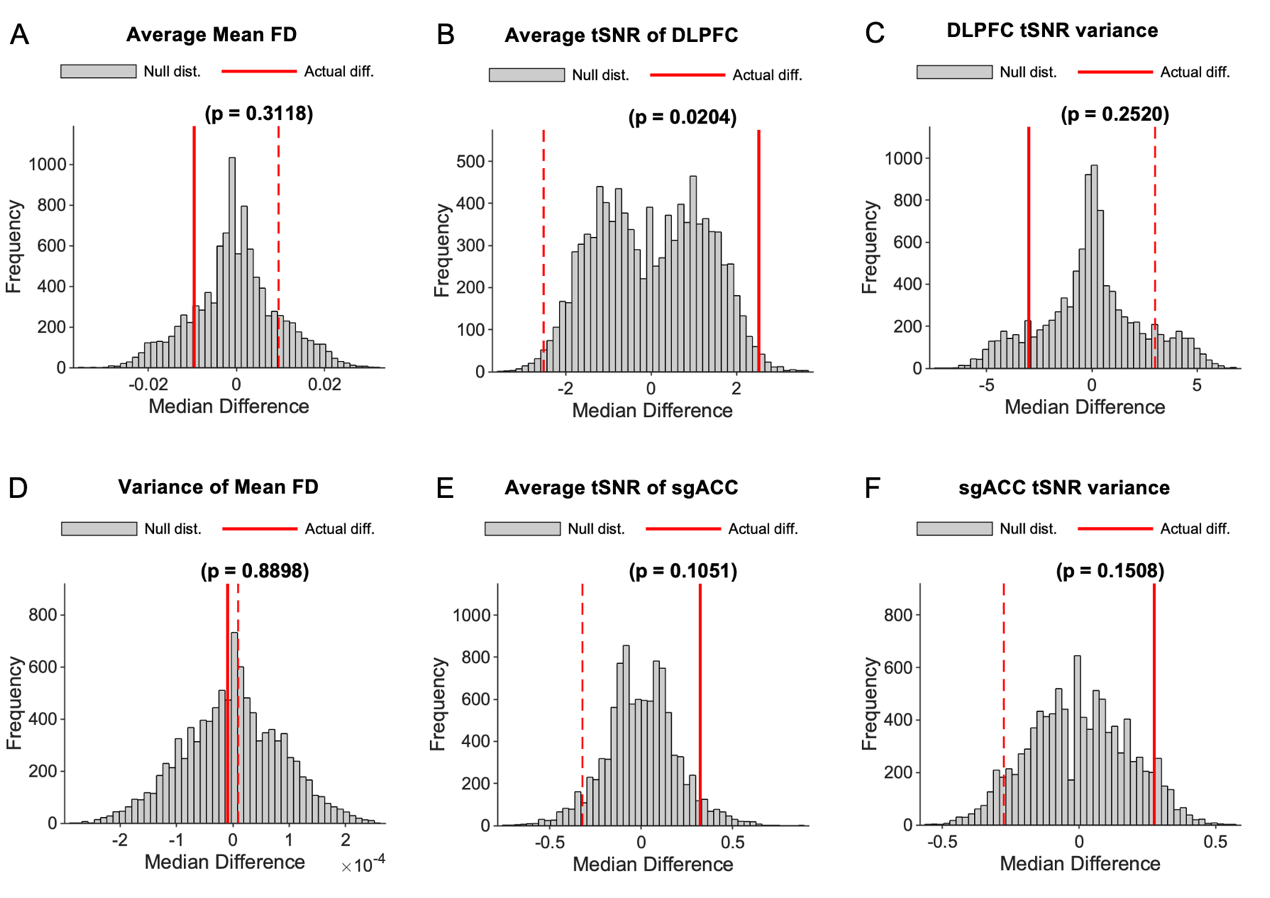


Figure S4. Comparison of Head Motion and Data Quality Metrics between RSA-defined Reliability Groups (high reliability vs. low reliability). (A) Mean FD averaged across 4 sessions, (B) Mean tSNR of DLPFC across 4 sessions, (C) Variance of tSNR within DLPFC across 4 sessions, (D) Variance of mean FD across 4 sessions, (E) Mean tSNR of sgACC across 4 sessions, (F) Variance of tSNR within sgACC across 4 sessions. The solid red line represents the true difference between the high‑ and low‑reliability groups. Abbreviations: FD, frame displacement; tSNR, temporal signal-to-noise ratio.


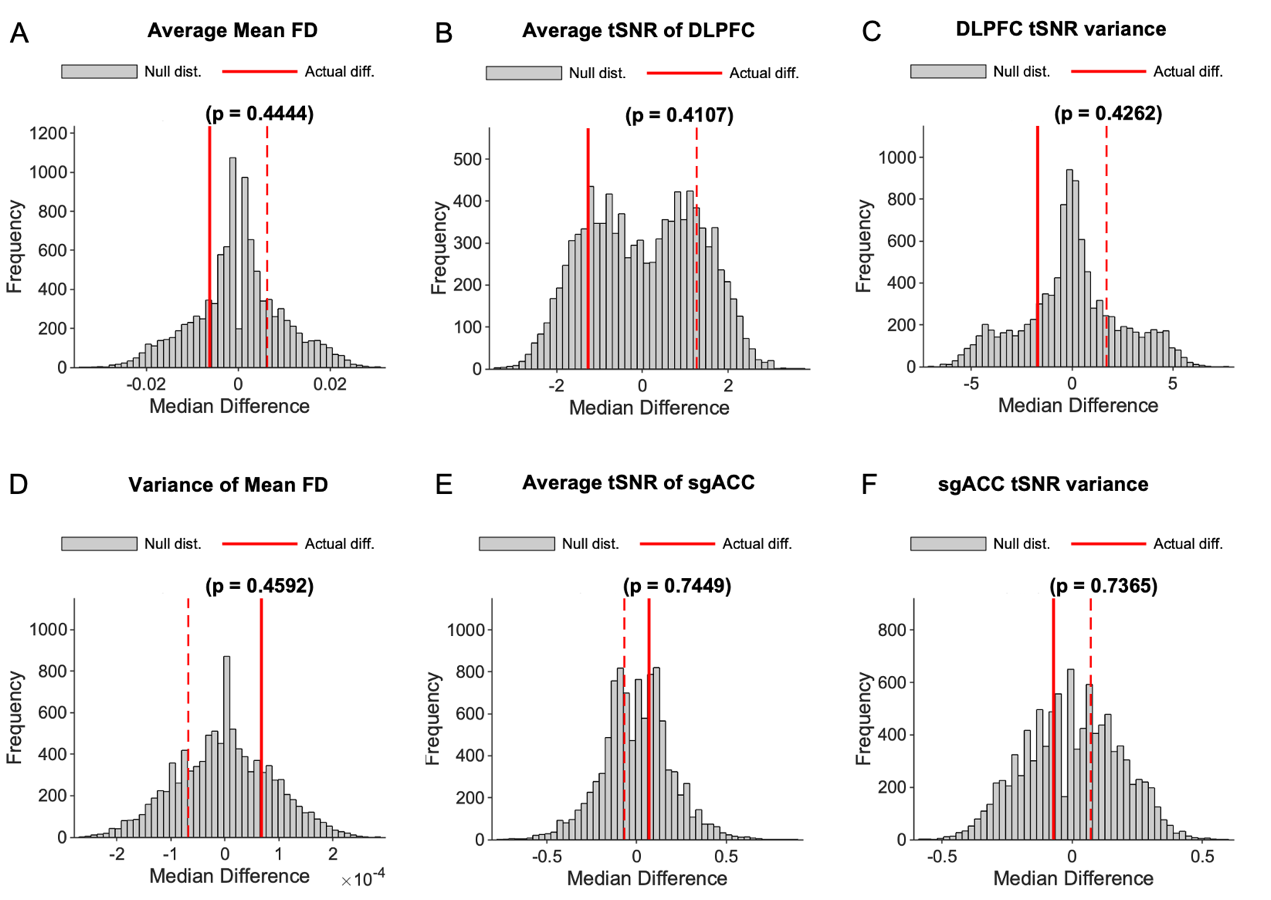


Figure S5. Comparison of Head Motion and Data Quality Metrics between HCA-defined Reliability Groups (high reliability vs. low reliability). (A) Mean FD averaged across 4 sessions, (B) Mean tSNR of DLPFC across 4 sessions, (C) Variance of tSNR within DLPFC across 4 sessions, (D) Variance of mean FD across 4 sessions, (E) Mean tSNR of sgACC across 4 sessions, (F) Variance of tSNR within sgACC across 4 sessions. The solid red line represents the true difference between the high‑ and low‑reliability groups. Abbreviations: FD, frame displacement; tSNR, temporal signal-to-noise ratio.

**Supplementary Analysis 2: Validation of Targeting Reliability in Clinical Populations and Impact of Data Length**

1. Validation in an Independent Clinical Dataset (TCP)

To evaluate the generalizability of the proposed algorithm, we conducted supplementary analyses using data from the Transdiagnostic Connectome Project (TCP) (OpenNeuro Accession Number: ds005237) (Chopra, Cocuzza et al. 2025). For this clinical validation, we selected participants who met the following stringent criteria: availability of four complete resting-state fMRI sessions, maximum head motion during any session not exceeding 3 mm in translation or 3° in rotation, and a clinical severity score of MADRS > 13. This selection process resulted in a final clinical cohort of 35 participants with MADRS scores ranging from 14 to 39, representing a spectrum from “mildly ill” to “severely ill” (Leucht, Fennema et al. 2017).

Test-retest reliability was evaluated using the Standard Deviation (SD) of distances (standard deviation of the Euclidean distances between the target location identified in each individual run and the centroid (mean location) of the four targets for that participant) and the averaged distance (across four runs). Consistent with the findings in the healthy HCP cohort, RSA demonstrated superior test-retest reliability, characterized by significantly lower SD and smaller averaged distances, compared to HCA when Global Signal Regression (GSR) was applied (Figure S6). In the absence of GSR, reliability metrics showed varying stability across different spatial axes, highlighting the influence of preprocessing choices on clinical functional connectivity patterns.

2. Controlling for the Impact of Data Length

Given that the TCP dataset has a shorter scan duration (488 time points) compared to the HCP dataset (1,200 time points), we investigated whether data length influenced the outcomes.

Specifically, we truncated the HCP data to the first 488 time points and recalculated the target stability. Even with the reduced data length, RSA consistently demonstrated significantly better test-retest reliability (lower SD and smaller average distance) than HCA, regardless of whether GSR was performed (Figure S6 C,D,E).

In conclusion, these supplementary analyses demonstrate that the reliability advantage of RSA is not limited to high-quality, long-duration healthy cohorts but extends to clinical depression populations.


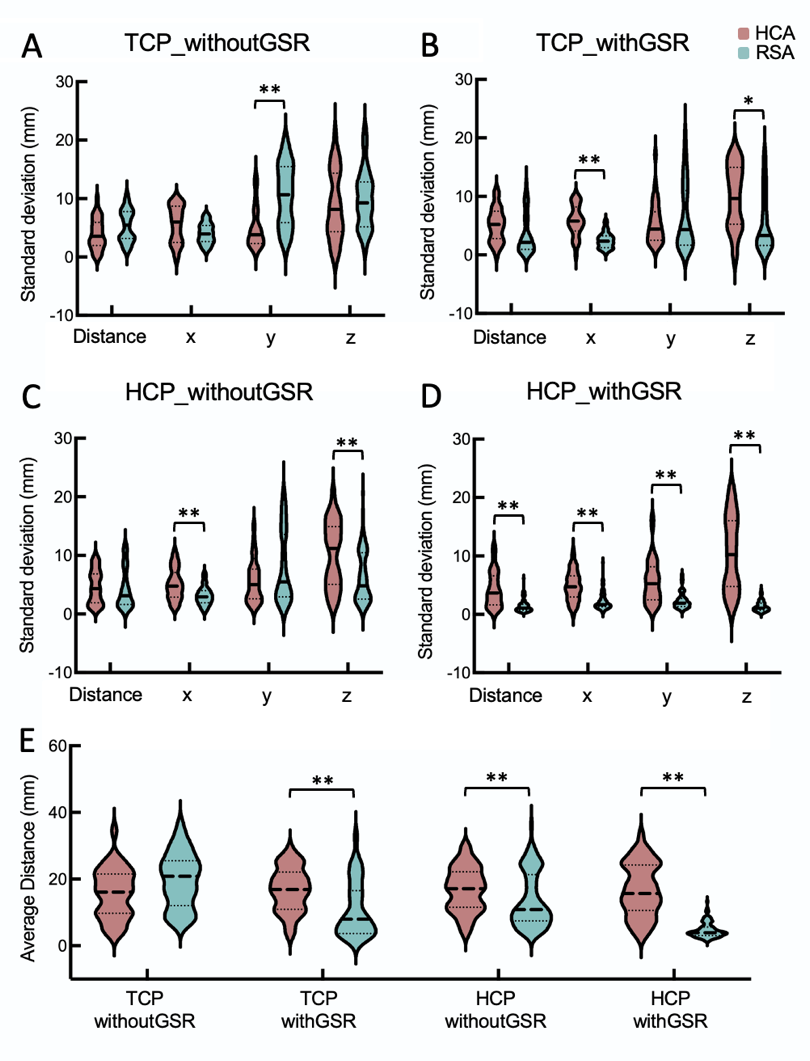


Figure S6. Test-retest reliability comparison results. Test–retest reliability was quantified using two complementary metrics: the standard deviation of 3D Euclidean distances and axis-specific (x, y, z) coordinate deviations for panels A–D, and the mean Euclidean distance across targets over four runs for panel E. Results are shown for the TCP dataset without global signal regression (GSR) (A) and with GSR (B), and for the HCP dataset (488 time points) without GSR (C) and after GSR (D). Panel E shows corresponding results (all for 488 time points) based on the mean distance metric.

Abbreviations: TCP, The Transdiagnostic Connectome Project; HCP, Human Connectome Project; GSR, global signal regression; HCA, hierarchical clustering algorithm; RSA, Refined seed-based algorithm. Note: * 0.01 < *p* < 0.05, ** *p* < 0.001. For each sub-figure, *p* < 0.025 was considered significant and was marked with stars.

Reference

Cash, R. F., L. Cocchi, J. Lv, Y. Wu, P. B. Fitzgerald and A. Zalesky (2021). "Personalized connectivity‐guided DLPFC‐TMS for depression: Advancing computational feasibility, precision and reproducibility." Human brain mapping **42**(13): 4155-4172.

Chopra, S., C. V. Cocuzza, C. Lawhead, J. A. Ricard, L. Labache, L. Patrick, P. Kumar, A. Rubenstein, J. Moses, L. Chen, C. Blankenbaker, B. Gillis, L. T. Germine, I. Harpaz-Rote, B. T. Yeo, J. T. Baker and A. J. Holmes (2025). Transdiagnostic Connectome Project. OpenNeuro.

Cole, E. J., K. H. Stimpson, B. S. Bentzley, M. Gulser, K. Cherian, C. Tischler, R. Nejad, H. Pankow, E. Choi and H. Aaron (2020). "Stanford accelerated intelligent neuromodulation therapy for treatment-resistant depression." American Journal of Psychiatry **177**(8): 716-726.

Fox, M. D., H. Liu and A. Pascual-Leone (2013). "Identification of reproducible individualized targets for treatment of depression with TMS based on intrinsic connectivity." Neuroimage **66**: 151-160.

Leucht, S., H. Fennema, R. R. Engel, M. Kaspers-Janssen, P. Lepping and A. Szegedi (2017). "What does the MADRS mean? Equipercentile linking with the CGI using a company database of mirtazapine studies." J Affect Disord **210**: 287-293.
